# A three-dimensional finite element model of round window membrane vibration before and after stapedotomy surgery

**DOI:** 10.1007/s10237-013-0479-y

**Published:** 2013-03-05

**Authors:** Monika Kwacz, Piotr Marek, Paweł Borkowski, Maciej Mrówka

**Affiliations:** 1 Faculty of Mechatronics, Institute of Micromechanics and Photonics, Warsaw University of Technology, ul. św. A. Boboli 8, 02-525 Warsaw, Poland; 2 Faculty of Power and Aeronautical Engineering, Institute of Aeronautics and Applied Mechanics, Warsaw University of Technology, ul. Nowowiejska 24, 00-665 Warsaw, Poland; 3 Institute of Physiology and Pathology of Hearing, ul. Mokra 17, 05-830 Nadarzyn, Poland

**Keywords:** Finite element model, Inner ear, Stapedotomy, Piston stapes prosthesis, Round window vibration

## Abstract

Piston stapes prostheses are implanted in patients with refractory conductive or mixed hearing loss due to stapes otosclerosis to stimulate the perilymph with varying degrees of success. The overclosure effect described by the majority of researchers affects mainly low and medium frequencies, and a large number of patients report a lack of satisfactory results for frequencies above 2 kHz. The mechanics of perilymph stimulation with the piston have not been studied in a systematic manner. The objective of this study was to assess the influence of stapedotomy surgery on round window membrane vibration and to estimate the postoperative outcomes using the finite element (FE) method. The study hypothesis is that the three-dimensional FE model developed of the human inner ear, which simulates the round window (RW) membrane vibration, can be used to assess the influence of stapedotomy on auditory outcomes achieved after the surgical procedure. An additional objective of the study was to enable the simulation of RW membrane vibration after stapedotomy using a new type of stapes prosthesis currently under investigation at Warsaw University of Technology. A three-dimensional finite element (FE) model of the human inner ear was developed and validated using experimental data. The model was then used to simulate the round window membrane vibration before and after stapedotomy surgery. Functional alterations of the RW membrane vibration were derived from the model and compared with the results of experimental measurements from temporal bones of a human cadaver. Piston stapes prosthesis implantation causes an approximately fivefold (14 dB) lower amplitude of the RW membrane vibrations compared with normal anatomical conditions. A satisfactory agreement between the FE model and the experimental data was found. The new prosthesis caused an increase of 20–30 dB in the RW displacement amplitude compared with the 0.4-mm piston prosthesis. In all frequencies, the FE model predicted a RW displacement curve that was above the experimental curves for the normal ear. The stapedotomy can be well simulated by the FE model to predict the auditory outcomes achieved following this otosurgery procedure. The 3D FE model developed in this study may be used to optimize the geometry of a new type of stapes prosthesis in order to achieve a similar sound transmission through the inner ear as for a normal middle ear. This should provide better auditory outcomes for patients with stapedial otosclerosis.

## Introduction

Stapedotomy surgery, first performed by Shea in 1956 (Shea 1958; Shea et al. 1962), is a commonly recognized, relatively safe and efficient surgical technique for certain types of auditory ossicle immobility, especially stiffening of the interface between the stapes footplate and the oval window, particularly in the course of otosclerosis (Rizer and Lippy 1993; Dubreuil et al. 1994; Häusler 2004; Somers et al. 2006;Vincent et al. 2006; Møller 2007). In stapedial otosclerosis, the stapes is immobilized by bone growth in the oval window niche. Fixation of the stapes footplate decreases its ability to vibrate, thereby significantly modifying the function of the normal ossicular chain, and always leads to conductive hearing loss (Dubreuil et al. 1994; Häusler 2004; Møller 2007). Stapedotomy surgery involves removing the superstructure of the otosclerotic stapes, drilling a small hole in the center of the immobilized stapes footplate, placing the piston of the stapes prosthesis (0.4–0.6 mm in diameter) into the hole, and tightening a wire or ribbon on the long process of the incus. The longitudinal vibration of the piston mimics the vibration of the normal stapes footplate, generates a pressure wave into the cochlea, and activates the sensory hair cells. The auditory outcomes obtained following the surgery should generally be perceived as good, but the good overclosure effect described by the majority of researchers mainly affects low (0.5 kHz) and medium frequencies (1 and 2 kHz) and a large number of patients report a lack of satisfactory results for frequencies above 2 kHz (Rizer and Lippy 1993; Häusler 2004; Somers et al. 2006; Vincent et al. 2006).

It has been shown (Wysocki et al. 2011) that the stapes piston prosthesis leads to an approximately 14 dB lower stimulation of perilymph vibration at RW compared to the physiological situation, in particular at frequencies exceeding 2 kHz. It has also been verified experimentally that the vibration amplitudes of the stapes piston prosthesis and the normal stapes are of the same order magnitude, while maintaining the same frequencies and sound pressure levels as the sounds supplied to the external auditory canal. This 14 dB lower stimulation of the perilymph may provide an explanation for the lack of the expected auditory outcome after stapedotomy surgery, especially for frequencies above 2 kHz.

Numerous clinical, experimental, and modeling studies have dealt with diagnosis of ear conditions, the design of implantable prostheses and hearing devices, and evaluation of devices or surgical treatment. In the modeling studies, several different methods are used to model hearing biomechanics.

Firstly, the analogy modeling method, represented by circuit or lumped parameter models based on acoustic-electrical analogues of the ear, has been developed and applied to predict the normal, pathological, and reconstructed behavior of both the middle (Møller 1961; Zwisłocki 1962; Shaw and Stinson 1981; Lynch et al. 1982; Kringlebotn 1988; Goode et al. 1994; Rosowski and Merchant 1995; Rosowski 1996; Feng and Gan 2004) and inner ear (Lyon and Mead 1988; Suesserman and Spelman 1993; Vanpoucke et al. 2004;Mistrík et al. 2009). Although these models are able to replicate experimental data, their parameters are not closely related to anatomical or physiological properties. Because of this, the analogy modeling method cannot be used to optimize the geometry of new types of middle ear prostheses.

Secondly, the oscillation of the cochlear partitions has been studied by analytical methods using two-dimensional and simplified three-dimensional models to investigate passive cochlear macro-mechanics, especially the vibration of the basilar membrane (Steele and Taber 1979; de Boer 1982; Kolston and Ashmore 1996; Parthasarati et al. 2000; Yoon et al. 2009). Mathematically, the macro-mechanical system of the cochlea can be described by fluid-mechanical equations coupled with equations describing the elastic properties of the BM, the bony walls of the SV and the ST, the RW membrane, and the oval window covered with elastic membrane. The mathematical problem of solving this system of partial differential equations on a three-dimensional domain is very difficult and requires application of advanced computational methods. In addition, the passive cochlear macro-mechanics cannot explain the extreme sensitivity and frequency selectivity of the basilar membrane. Since the discovery of outer hair cells (OHCs) electromotility (Brownell et al. 1985), cochlear modeling has been focusing on exploring active process and nonlinear behavior in the BM responses measured in the live cochlea. A number of active cochlear models have introduced OHC motility into cochlear macro- and micromechanics (Kolston et al. 1989; Hubbard 1993; Neely 1993; Geisler 1993; Geisler and Sang 1995; Fukazawa 2002; Lim and Steele 2002; Shatz 2005; Yoon et al. 2009, 2011). The nonlinear models were either solved in the time domain or in the frequency domain using iterative or perturbation techniques. Our purpose, however, is not to study the active behavior of the cochlea, but to predict the auditory outcomes achieved following stapedotomy surgery with various types of stapes prostheses.

Finally, finite element (FE) methods have been applied to study the function of the normal or reconstructed human middle (Wada et al. 1992; Funnell 1996; Ferris and Prendergast 2000; Daniel et al. 2001; Kelly and Prendergast 2001; Gan et al. 2002, 2004; Gan et al. 2007, 2009; Gan et al. 2010; Gil-Carcedo et al. 2002; Koike et al. 2002; Sun et al. 2002; Kelly et al. 2003; Böhnke and Arnold 2007; Zhao et al. 2009; Zhang and Gan 2011) and inner ear (Böhnke and Arnold 1999; Hanekom 2001; Hanekom 2005; Kiefer et al. 2006; Ramamoorthy et al. 2007). The FE method is a numerical technique commonly used to model mechanical behavior of complex biological systems. This technique is especially useful for simulating the behavior of structures in conditions that cannot be achieved experimentally and is suitable for modeling the complex geometry and anisotropic material properties of biological systems. The first FE model of the cat tympanic membrane was reported by Funnell and Laszlo (1978). Since then, FE modeling of middle and inner ear dynamic has become a fast growing research area in the study of auditory mechanics. The FE models were used to simulate the detailed vibration shapes, stress distributions, and dynamic behaviors at any location in the hearing organ. In order to create a useful model that can simulate the functional behavior of the inner ear, most of the authors simplify the complex cochlear anatomy, especially often its coiled shape, and approximate the dimensions, boundary conditions, and material properties of cochlear tissues. Recently, there have been two major reasons for developing FE models – one is to simulate the acoustic-mechanical transmission from the external auditory canal to the cochlea with a focus on the middle ear’s transfer function (Koike et al. 2002; Gan et al. 2004, 2006; Gan et al. 2007, 2009; Gan and Wang 2007; Zhao et al. 2009). The other is to simulate the pressure wave traveling along the basilar membrane from the oval window to the apex in the cochlea with a focus on cochlear mechanics (Gan et al. 2007; Gan and Wang 2007; Böhnke and Arnold 2007).

Despite a range of modeling studies, there is still limited information on sound transmission through the inner ear after stapedotomy surgery, mainly due to an insufficient number of experimental and theoretical studies. The aim of this study is to assess the influence of various types of stapes prostheses on the round window membrane vibration and estimate the postoperative outcomes using the finite element (FE) method. By comparing the response of the normal ear to that of the ear with prosthesis inserted, it is expected that advances can be made in improving the design of a new stapes prosthesis.

### Objective of the study

The study hypothesis is that the three-dimensional FE model developed for the human inner ear, which simulates the round window (RW) membrane vibration determining the intensity of energy transmission from the outer ear to the inner ear compartments, can be used to assess the influence of stapedotomy on auditory outcomes achieved after the surgical procedure. Differences in the magnitudes of vibration amplitude were expected to occur, particularly for different sound frequencies. This could confirm clinical observations indicating variable auditory outcomes of stapedotomy for individual frequencies.

An additional objective of the study was to enable the simulation of RW membrane vibration parameters after stapedotomy using a new type of stapes prosthesis currently under investigation at Warsaw University of Technology. The parameters calculated may be used to optimize the geometry of the prosthesis in order to achieve a similar intensity of sound transmission as in the case of the normal middle ear. This should provide better auditory outcomes for patients with stapedial otosclerosis.

## Materials and methods

The modeling studies were conducted using a 3D finite element model of the uncoiled cochlea and consisted of simulation of the RW membrane vibration amplitude in the frequency range 0.4–10 kHz in both the pre-stapedotomy and post-stapedotomy states. In the post-stapedotomy state, the differences in the RW membrane displacement amplitudes were studied for two piston prostheses of different diameters (0.4 and 0.6 mm). The vibrations of the RW membrane obtained from the FE model were subsequently compared with the results of experimental measurements in human temporal bones using the scanning laser Doppler vibrometer (SLDV PSV 400 system, Polytec GmbH, Waldbronn, Germany).

In addition, by using the model developed, round window membrane vibrations were predicted after implantation of a new type of stapes prosthesis.

### 3D finite element model of the inner ear/geometry and mesh of the inner ear structures

#### The pre-stapedotomy model

The three-dimensional FE model of the uncoiled cochlea consists of two straight fluid channels representing the scala vestibuli (SV) and scala tympani (ST), separated by the basilar membrane (BM). The third channel (scala media) was included in the SV. The Reissner’s membrane and the micromechanical structures of the organ of Corti were not considered in the model. The stapes footplate (SF) was attached to the SV in the oval window by an annular ligament (AL) and the ST was connected to the middle ear cavity at the round window niche by the RW membrane. The SV and ST were filled with an viscous perilymph fluid. At the apex region, these two channels were connected to each other through the helicotrema.

**Fig. 1:**
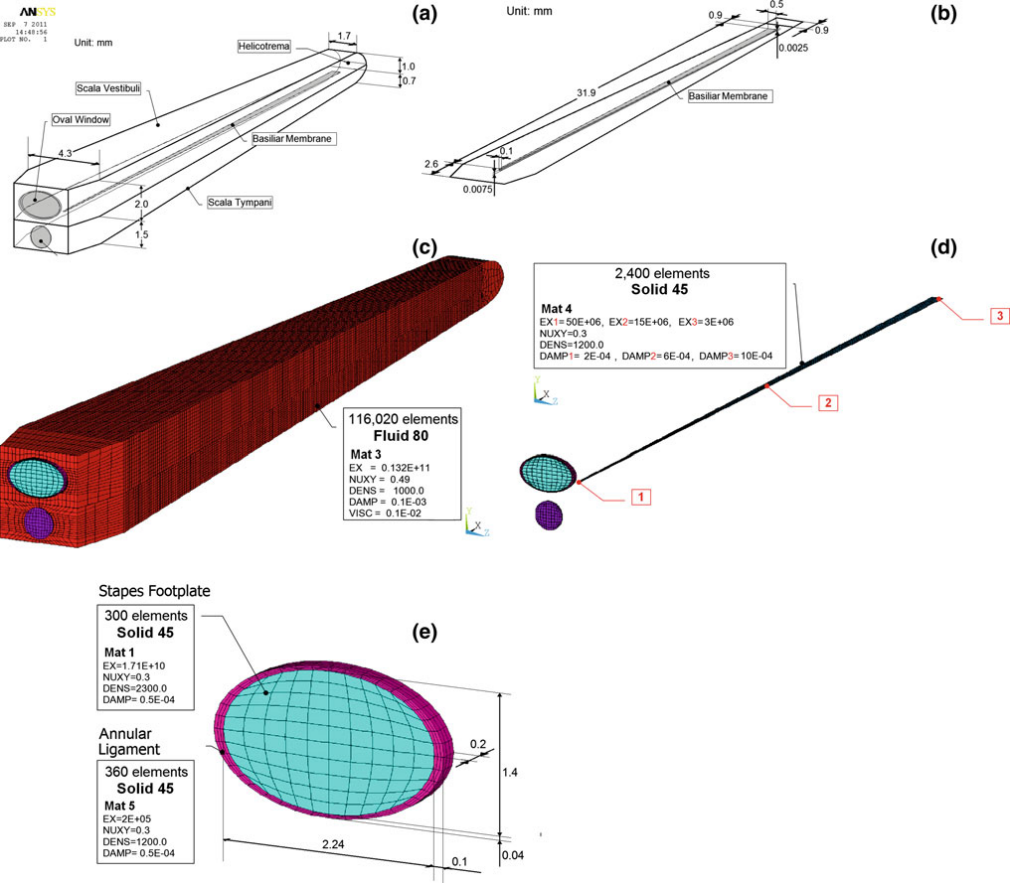
The structure of the simplified cochlear model with detailed dimensions and FE meshes of the anatomical structures in the pre-stapedotomy FE state. **a** 3D schematic view of the uncoiled cochlea showing the overall dimensions of the scala vestibule (SV) and the scala tympani (ST), **b** 3D view of the basilar membrane (BM) and the supported structures of the BM with detailed dimensions of the BM, **c** 3D view of the uncoiled cochlea showing the FE mesh of both the SV and the ST with the mechanical properties assumed in the FE model, **d** 3D view of the BM with the mechanical properties assumed in the FE model, 1—at the base, 2—in the middle, 3—at the apex, **e** 3D view of the stapes footplate (SF, *blue*), the annular ligament (AL, *pink*), and the round window membrane (RW, *violet*) showing the detailed dimensions, the FE meshes, and the mechanical properties of these three structures

The geometry of the inner ear structures was adopted according to dimensions published in the literature (Gan et al. 2007), similar to the real coiled geometry of the cochlea. Our FE model was constructed based on the information given by Gan’s group, but these two models differ from each other. The main differences are the geometry of the stapes footplate, the annular ligament, the RW membrane, the variable height of both SV and ST, the finite element mesh and number of finite elements for the individual structures, the material properties of the stapes footplate and the annular ligament, except density of the AL. The geometry was then meshed in ANSYS 13.0 (Ansys, Inc., Canonsburg, PA). Figure 1 shows the structure of the simplified cochlear model with detailed dimensions and FE meshes of the anatomical structures.

The fluid-filled SV and ST channels were assumed to change linearly in height from 2.0 mm at the base to 1.0 mm at the apex and from 1.5 mm at the base to 0.7 mm at the apex, respectively. The length of both the SV and ST was assumed to be 33.8 mm and the width was assumed to change linearly from 4.3 mm at the base to 1.7 mm at the apex. The helicotrema opening at the apex was modeled as a rectangular fluid passageway with dimensions of $1.7\times 1.8$ mm. The SV and the ST channels with the helicotrema were filled with a viscous fluid and meshed by 116020 eight-node hexahedral fluid elements (Fluid80 in ANSYS). The Fluid80 elements at a boundary are not directly attached to the structural Solid45 elements (of the BM, the RW membrane, the SF, and the AL) but have separate, coincident nodes that are coupled only in the direction normal to the interface.

The BM dimensions were assumed to change linearly in width from 0.1 mm at the base to 0.5 mm at the apex and in thickness from 7.5 $\upmu$m at the base to 2.5 $\upmu$m at the apex. The length of the BM was assumed as 31.9 mm. The BM was meshed by 2400 eight-node 3D hexahedral solid elements (Solid45 in ANSYS). The boundary conditions for the BM were defined as fully clamped (all displacements and rotations zero) at the spiral lamina and simply supported (all displacement zero) at the spiral ligament. This is motivated by Iurato’s anatomical studies (Iurato 1962), in which the main supporting bundles of the spiral lamina continue directly into the fibers of the BM, suggesting a clamped condition. On the other side of the BM, the fibers continue directly into the spiral ligament but suddenly become thinner prior to joining the spiral ligament, suggesting a simply supported boundary condition.

The SF was assumed to be an ellipse with a long axis of 2.24 mm and a short axis of 1.4 mm. The AL was assumed to be an elliptical ring with a width changing from 0.1 mm in the long axis to 0.04 mm in the short axis. The thickness of both the AL and the SF was assumed to be 0.2 mm. The RW membrane was assumed as a circle with a diameter of 1.2 mm and a thickness of 0.05 mm. All nodes along the outer perimeters of both the AL and the RW membrane were fixed. The nodes along the inner perimeter of the AL were connected with the nodes along the outer perimeter of the SF. The AL, the SF, and the RW membrane were meshed by 360, 300, and 300 eight-node 3D hexahedral solid elements (Solid45), respectively. The FE mesh has been adopted taking into account a trade-off between computational time and accuracy. To establish that the mesh was fine enough, we have performed a series of simulations with different mesh density until the simulation results no change by more than 5 %. The accuracy of simulation results was defined as the correlation between the model-derived and experimental magnitudes of the RW membrane displacements in the pre-stapedotomy state. Correlation analysis (when the AL, the SF, and the RW membrane were meshed by 360, 300, and 300 elements, respectively) between the model and experimental results for frequencies $<$8 kHz was significant except the correlation at 0.63 kHz (Pearson correlation coefficient $r = 0.91$, $p < 0.05$). Figure 5 shows the experimental and model-derived curves obtained for the accepted FE mesh. Differences between the model results and experiments at 8 kHz and 10 kHz were described in the chapter Discussion.

**Fig. 2:**
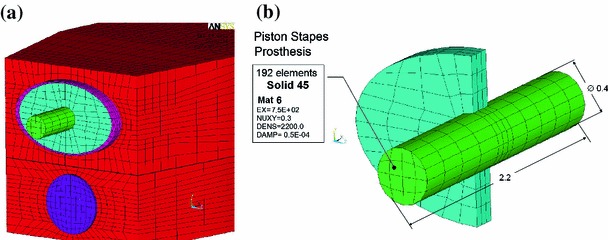
The structure of the simplified cochlear model in the post-stapedotomy state. The geometry, boundary conditions, and FE meshes of the other elements of the post-stapedotomy model were *left* the same as in the pre-stapedotomy model. **a** 3D schematic view showing the shape and the location of the piston stapes prosthesis (*green*) in the stapes footplate (*blue*), **b** 3D view of the stapes footplate and the piston of the prosthesis showing the dimensions, the FE mesh, and the mechanical properties of the piston and the modified FE mesh of the stapes footplate

**Table 1:** Mechanical properties of the ear structures used in the FE inner ear model

Structure	Data used in the FE model	Source
*Stapes footplate (SF)*		
Density (kg/m$^{3}$)	2.30 E3	The FE model
Young’s modulus (N/m$^{2}$)	1.71 E10	The FE model (determined by the FE model validation process)
Damping	$\alpha =0\,\text{ s}^{-1}$, $\beta = 0.00005$ s	The FE model (determined by the FE model validation process)
*Annular ligament (AL)*		
Density (kg/m$^{3}$)	1.20 E3	Gan et al. 2007
Young’s modulus (N/m$^{2}$)	2.00 E5	Gan 2006
Damping	$\alpha = 0\,\text{ s}^{-1},\,\beta$ = 0.00005 s	The FE model (determined by the FE model validation process)
*Round window membrane (RW)*		
Density (kg/m$^{3}$)	1.20 E3	Gan et al. 2007
Young’s modulus (N/m$^{2}$)	3.50 E5	Gan et al. 2007
Damping	$\alpha = 0 \text{ s}^{-1}$, $\beta$ = 0.00005 s	Gan et al. 2007
*Basilar membrane (BM)*		
Density (kg/m$^{3}$)	1.20 E3	Gan et al. 2007
Young’s modulus (N/m$^{2}$)		
At the base	5.00 E7	Gan et al. 2007
In the middle	1.50 E7	Gan et al. 2007
At the apex	3.00 E6	Gan et al. 2007
Damping		
At the base	$\alpha = 0 \,\text{ s}^{-1},\, \beta$ = 0.0002 s	Gan et al. 2007
In the middle	$\alpha = 0\, \text{ s}^{-1},\, \beta$ = 0.0006 s	Gan et al. 2007
At the apex	$\alpha = 0 \,\text{ s}^{-1},\, \beta$ = 0.001 s	Gan et al. 2007
*Perilymph fluid*		
Density (kg/m$^{3}$)	1.00 E3	Gan et al. 2007
Bulk modulus (N/m$^{2}$)	2.2 E9	Gan et al. 2007
Damping	$\alpha = 0.001\, \text{ s}^{-1},\, \beta$ = 0.0001 s	Gan et al. 2007

#### The post-stapedotomy model

To assess the effect of stapedotomy on sound transmission into the inner ear, the pre-stapedotomy model was modified as follows: the SF was immobilized by complete fixation of the AL nodes, a hole with a diameter of 0.4 mm was made in the center of the SF, and the piston of the stapes prosthesis with the same diameter was placed into the hole. The piston of the stapes prosthesis was assumed as a cylinder with a diameter of 0.4 mm and a length of 2.2 mm (Fig. 2). The piston was meshed by 192 eight-node 3D hexahedral solid elements (Solid45 in ANSYS). The geometry, boundary conditions, and FE meshes of the other elements of the post-stapedotomy model were left the same as in the pre-stapedotomy model.

### Material properties of the inner ear structures

The material properties of the inner ear structures were initially assumed according to the properties published in the literature. These parameters were then checked and some of them modified in the validation process. The mechanical properties of the inner ear structures used in the inner ear model, including the density, Young’s modulus, and Raleigh damping parameters $\alpha$ and $\beta$ of the SF, the piston of the stapes prosthesis, the AL, the RW membrane, and the BM, are listed in Table 1.

All solid materials, except the BM, in both the pre-stapedotomy and post-stapedotomy models were assumed as isotropic materials with the Poisson’s ratio of 0.3 and the damping coefficient $\beta$ of 0.5E-04 s. The Young’s moduli of the SF, the piston of the stapes prosthesis, the AL, and the RW membrane were assumed as 1.71E+04, 750, 0.2, and 0.35 MPa, respectively. The density of the structures mentioned above was assumed as 2,300, 2,200, 1,200, and 1,200 kg/m$^{3}$, respectively.

The BM was treated as a material with a Poisson’s ratio of 0.3 and a density of 1,200 kg/m$^{3}$. We assumed the Poisson’s ratio of 0.3 after Gan et al. (2007; p. 2182). The effect of the Poisson’s ratio on the BM membrane deflection was checked by Liu and White (2008). In Fig. 4, they showed that the choice of this parameter (0.2 or 0.4) does not have a major impact on vertical displacement of the BM. It is well known that the BM stiffness varies along its length (Emadi et al. 2004; Liu and White 2008). According to the data used in a 3D cochlea model by Gan et al. (2007, 2009), the Young’s modulus of the BM was assumed to change from 50 MPa at the base to 3 MPa at the apex. Having determined the Young’s modulus value of the BM, the damping in the FE model was then adjusted so that the derived RW responses matched with both our experimental results and data from the literature (Asai et al. 1999; Stenfelt et al. 2004a, b). The damping was adjusted by varying the material damping factor ($\beta$) of the BM. While adjusting the BM damping, the BM responses were also checked. The damping coefficient $\beta$ of the BM was finally assumed to change from 0.2E-03 s at the base to 0.1E-02 s at the apex.

The perilymph fluid filling the SV and ST in the cochlea was assumed as a viscous fluid with a density of 1,000 kg/m$^{3}$. The viscosity of the fluid was assumed to be 0.001 Ns/m$^{2}$.

### Finite element analysis

Structural and fluid FE analyses were performed in ANSYS 13.0 (ANSYS, Inc., Canonsburg, PA) to determine the displacement amplitudes of the RW membrane at different frequencies, from both the pre-stapedotomy and the post-stapedotomy model.

The motion of perilymph fluid in the cochlea is assumed to be described by the continuity equation for small-compressible flow:

$$\begin{aligned} P=-k\nabla \cdot {\varvec{u}} \end{aligned}$$

where $P$ is pressure, *** u*** is the displacement vector, $k$ is the bulk modulus of fluid, and $k=c^{2}\,\rho$ . In this study, the coefficient $k$ was assumed to be 2.20 GPa.

The harmonic analysis was conducted within the auditory frequency range of 0.4–10.0 kHz. The displacement excitation was applied at the nodes of either the SF or the piston prosthesis along the direction perpendicular to the footplate. The magnitudes of the displacement amplitudes were assumed to be exactly the same as the magnitudes measured for the human temporal bones in our experiment using a scanning laser Doppler vibrometer.

In this study, the investigations were conducted using a two-step scheme.

Firstly, the FE analysis from the pre-stapedotomy model was employed to determine the displacement–frequency curve of the RW membrane and to adjust and validate the model. In the model validation process, the harmonic displacement excitation was applied to the surface of the SF, the vibration response of the RW membrane was calculated, and the values of the model parameters were estimated to achieve satisfactory agreements between the model and experimental data.

Secondly, the FE analysis of the post-stapedotomy model was employed to investigate the effect of piston prosthesis insertion on the RW membrane vibration using the validated FE model.

### Measurement in human temporal bones

Four isolated human temporal bones, three males and one female, selected at the Forensic Medicine Institute of the Warsaw Medical University were included in this study. The donors’ ages were 18, 27, 29, and 37 years.

The methods for temporal bone preparation, the experimental stapedotomy procedure, acoustic and optical pathway structure and calibration, and round window vibration patterns before and after stapedotomy have already been detailed elsewhere (Wysocki et al. 2011).

In brief, human cadaver temporal bones were prepared by shortening the bony ear canal to approximately 1 cm, placing a polyurethane foam ear tip (ER3-14A, Etymotic Research, Elk Grove Village, IL) in it, performing a maximally wide posterior tympanotomy, and a wide approach in the RW niche through the jugular fossa. Sound stimulation was introduced to a sealed ear canal from an insert earphone (ER-2, Etymotic Research) via a flexible sound delivery tube with adapter (ER1-21, Etymotic Research). The ear canal sound pressure level (SPL) was recorded with a calibrated probe tube microphone (ER-7C Etymotic Research) with the tip of the flexible probe tube (ER7-14C, Etymotic Research) positioned approximately 2 mm distant from the tympanic membrane. Based on the SPL value measured by the microphone, we corrected the amplification of the input acoustic signal supplied to the loudspeaker to maintain a constant SPL value of 90 dB for each of the measurement frequencies in a 0.4–10 kHz frequency range.

At the first stage of measurement (the pre-stapedotomy state), the vibration amplitudes of both the RW and the stapes were recorded in normal anatomical conditions. Then, in the same specimen, the stapes footplate was immobilized in the OW niche with glass ionomer cement and the experimental stapedotomy procedure was performed with a standard Teflon piston stapes prosthesis (DEMED$^{\circledR }$, Mikołów, Poland). Two Teflon piston prostheses of different diameters (0.4 and 0.6 mm) were used successively. Immediately after the piston with a diameter of 0.4 mm was inserted, another series of vibration amplitudes of both the RW and the prosthesis was recorded (post-stapedotomy state 1). Afterward, in the same specimen, the hole was enlarged, the other prosthesis with a piston diameter of 0.6 mm was placed into the hole, and the last series of the vibration amplitudes of both the RW and the prosthesis was recorded (post-stapedotomy state 2).

The stapes, stapes prosthesis, and RW displacements were measured with two scanning laser Doppler vibrometers (PSV400, Polytec GmbH, Waldbronn, Germany). One laser was directed through the posterior tympanotomy at the stapes for the pre-stapedotomy state or at the piston for the post-stapedotomy state. The deviation of this laser beam varied 25$^{\circ }$–40$^{\circ }$ with respect to the direction of the stapes movement (i.e., the direction perpendicular to the plane of the stapes footplate). The other laser was directed through the jugular fossa at the RW membrane for both the pre-stapedotomy and post-stapedotomy state. The deviation of the laser beam with respect to the direction of RW membrane movement varied 15$^{\circ }$–35$^{\circ }$. The deviation angles of the laser beams were used as a cosine correction factor to obtain the real displacement amplitudes of the measured structures.

The vibrometer controller was programmed to generate a succession of acoustic input signals with a center frequency of successive one-third octave bands: 0.40, 0.50, 0.63, 0.80, 1.00, 1.25, 1.60, 2.00, 2.50, 3.15, 4.00, 5.00, 6.30, 8.00, and 10.0 kHz. For each measurement frequency, the sound pressure level was maintained at the same value of 90 dB.

The displacement amplitudes of the stapes head and piston prosthesis obtained from the measurements were then used in our FE model as amplitudes of the harmonic displacement excitations for the pre-stapedotomy and post-stapedotomy model, respectively. The experimental displacement–frequency curve of the RW membrane for the pre-stapedotomy state was then used to validate the FE model and the curves for the post-stapedotomy state as control data.

## Results

### Displacement excitation A_in

**Fig. 3:**
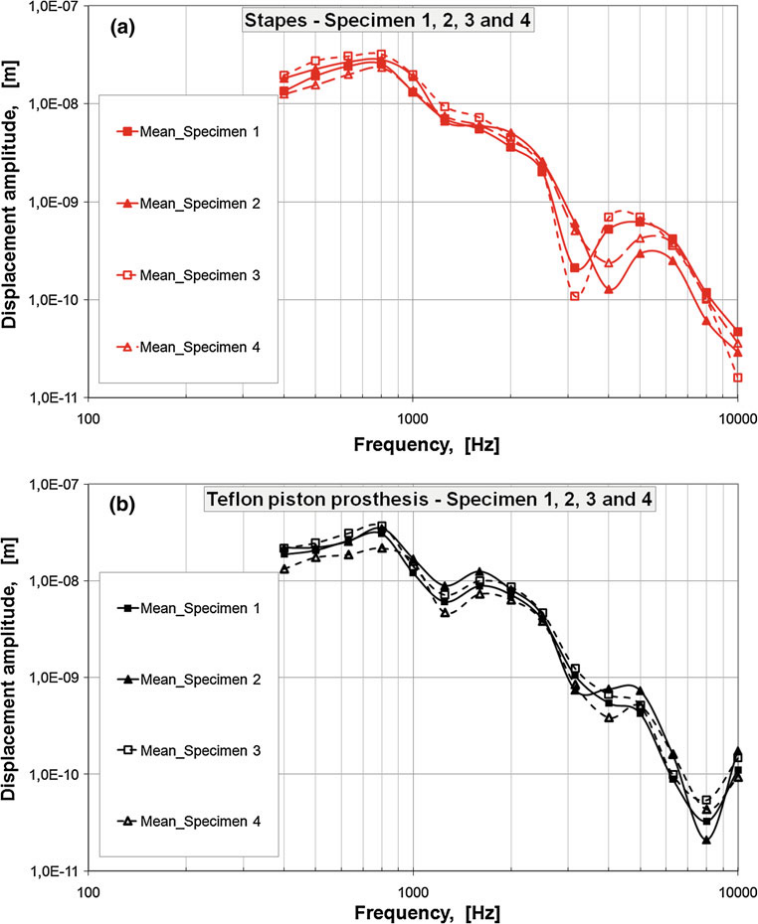
Displacement amplitudes of **a** stapes head and **b** stapes piston prosthesis for four temporal bones. The mean values were determined for 73 scan points on the stapes head and 38 scan points on the piston prosthesis. The specimens were stimulated with a sound pressure level of 90 dB SPL at the tympanic membrane

The vibration responses of the SF and piston stapes prosthesis with air conduction (AC) stimulation were measured in four temporal bone specimens using a scanning LDV system. Figure 3 presents the results (the mean values determined for 73 scan points on the stapes head and 38 scan points on the piston prosthesis) from all four specimens. In this study, we analyzed the experimental and model-derived amplitude frequency profiles for Specimen 1. The pre- and post-stapedotomy profiles for all measurement points from Specimen 1 were presented in Kwacz et al. (2012, Figure 2).

**Fig. 4:**
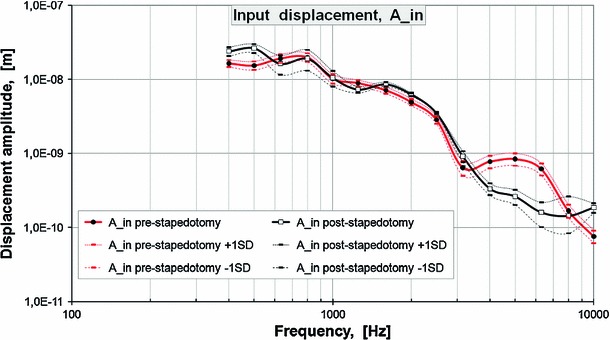
Pre-stapedotomy and post-stapedotomy experimental input displacement amplitude (A_in) frequency profiles assumed as the amplitudes of the harmonic displacement excitations in the pre-stapedotomy and post-stapedotomy FE models. The figure presents the average values of displacement amplitude for specimen 1 (A_in, *thick, solid curve line*) with standard deviation $\pm$1 SD markers jointed by a *thin, dashed curve line*. The average and the standard deviation were calculated for each measurement frequency based on the 73 pre-stapedotomy and the 38 post-stapedotomy amplitude frequency profiles showed in Kwacz et al. (2012, Fig. 2)

Based on the experimental amplitude frequency profiles, the input displacement amplitude A_in at each measurement frequency was determined as the average value of the displacement amplitudes measured on the surface of the stapes head (pre-stapedotomy state) and piston stapes prosthesis (post-stapedotomy state). The pre-stapedoptomy and post-stapedotomy input displacement amplitudes for specimen 1 are shown in Fig. 4 and in Table 2. These displacement amplitudes A_in were assumed as the amplitudes of the harmonic displacement excitations in the pre-stapedotomy and post-stapedotomy FE model.

### Validation of the FE model

The FE model of a normal cochlea (pre-stapedotmy model) was first validated by comparing the displacement–frequency curves of the RW membrane derived from the model with the experimental data obtained from human temporal bones.

In the validation process, the initial displacements were assumed as harmonic displacements of A_in pre-stapedotomy amplitudes (Table 2) and applied to all nodes of the normal SF in the direction perpendicular to the footplate. Then, the magnitudes of the RW membrane displacements for the central point on the RW membrane were calculated from the FE model for each measurement frequency.

The experimental displacement–frequency curves of the RW membrane were reported in our previous paper (Wysocki et al. 2011). It should be noted that only four points at the center of the RW membrane were considered for validation. For these four points, the average value of RW membrane displacement was calculated and adopted for comparison with the model-derived value for each measurement frequency.

**Fig. 5:**
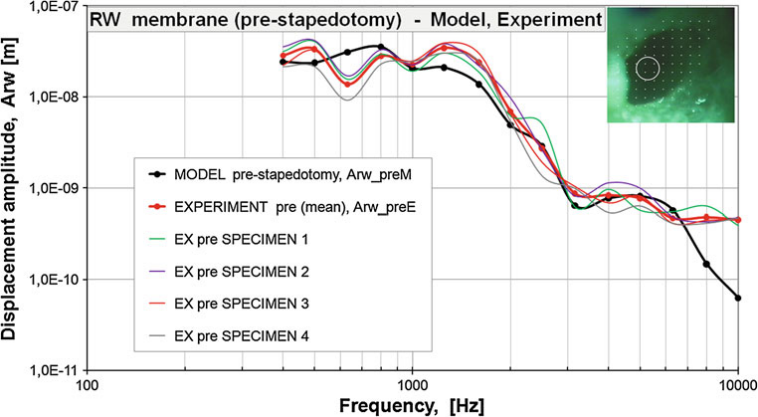
Pre-stapedotomy amplitude frequency profiles of the RW membrane displacement. The figure presents the model-derived frequency response curve (MODEL pre-stapedotomy, *thick, black, solid curve line*) while the amplitudes of the harmonic displacement excitations are given in Table 2 (A_in pre-stapedotomy) in comparison with the corresponding curves (EX pre SPECIMEN, *thin, colored, solid curve lines*) obtained from four temporal bone specimens in the pre-stapedotomy state when 90 dB SPL pure tones were applied in the external ear canal and values of the average displacement amplitude of four points for all specimens (EXPERIMENT pre(mean), *thick, gray, dashed curve line*)

Figure 5 shows the model-derived frequency response curve of the RW membrane displacement in comparison with the corresponding curves obtained from four temporal bone specimens in the pre-stapedotomy state when 90 dB SPL pure tones were applied in the external ear canal.

As can be seen in Fig. 5, the FE model-predicted RW displacement curve falls within the range of the four temporal bone experimental curves across the frequency range of 0.5–7 kHz. However, there are some discrepancies between the model and experimental results. The displacement magnitudes from modeling at high frequencies ($f>7$ kHz) lie below the experimental curves. The model did not show either the pronounced peak at 1.25 kHz or the minimum at 0.63 kHz that were seen experimentally in all specimens. At frequencies of 1–2 kHz, the FE model curve is slightly lower than the mean experimental curve. However, the trend of the FM model-predicted RW displacement curve is similar to the mean experimental curve.

**Fig. 6:**
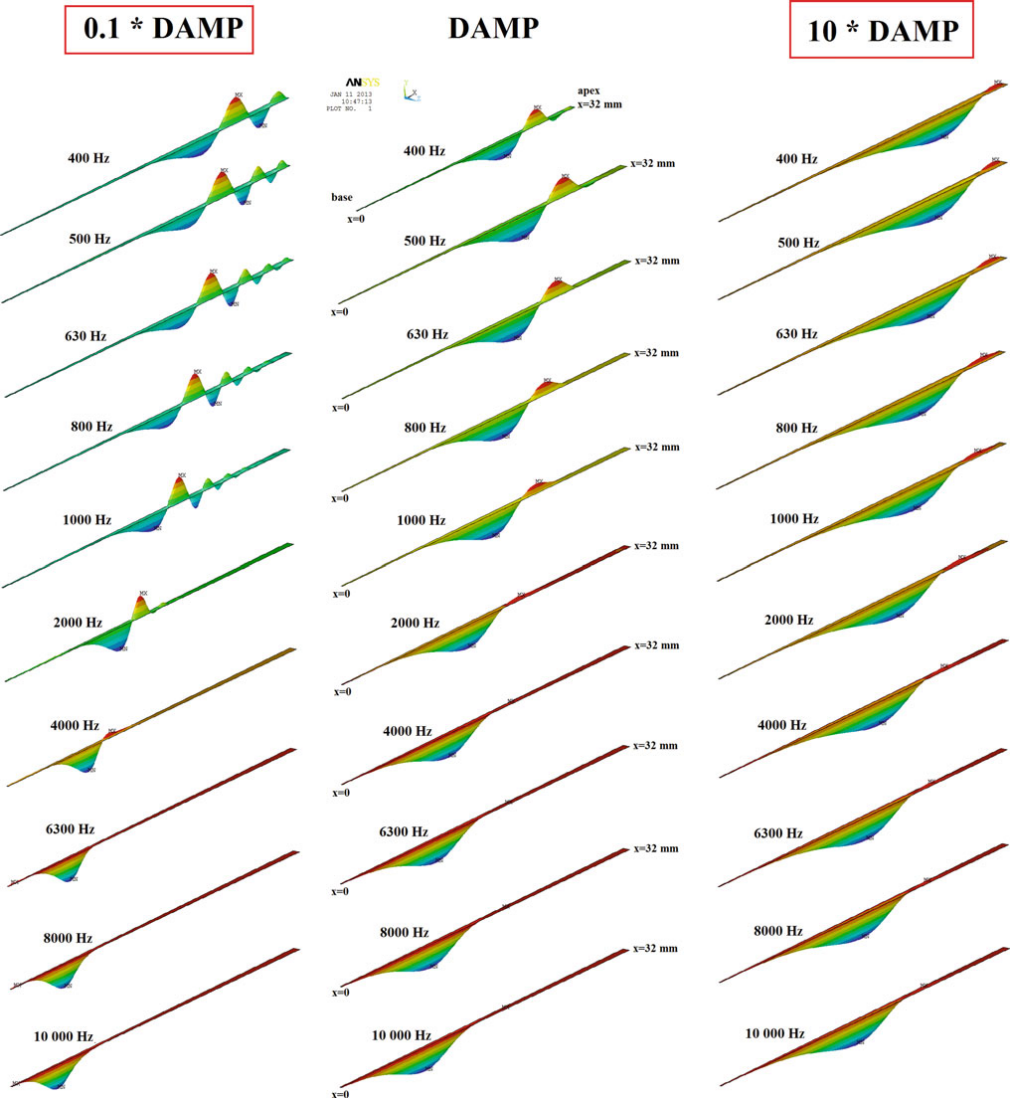
The traveling wave pattern along the BM from the base ($x=0$) to the apex ($x=32$ mm) at frequencies of 0.4–10 kHz for different values of the BM damping coefficient $\beta$ (0.1*DAMP, DAMP, 10*DAMP, where DAMP changes linearly from 0.2E-03 s at the base to 0.1E-02 s at the apex)

Figure 6 shows the model-derived BM responses obtained for different values of the BM damping coefficient $\beta$ (0.1*DAMP, DAMP, 10*DAMP, where DAMP changes linearly from 0.2E-03 s at the base to 0.1E-02 s at the apex). As can be seen, the damping coefficient affects decay of the BM amplitude past the first peak. For $\beta =0.1$*DAMP, the secondary peaks of the BM amplitude are observable for frequencies 0.4–2 kHz. Moreover, the maximum BM displacement for 2 kHz is more basally compared with data reported by Georg von Bekesy. This indicates that the damping coefficient of $\beta =0.1$*DAMP is too small. For $\beta =\text{ DAMP}$, the secondary peaks are significantly reduced and for $\beta =10$*DAM almost not observable. Increase in the BM damping also shifts the maximum BM displacement toward the apex. The simulation results have also shown that the change in the damping coefficient $\beta$ affects only on the BM motion and the RW motion remains the same. Therefore, we claim that the BM damping coefficient should be assumed as DAMP or greater.

**Table 2:** Amplitude of the harmonic displacement excitations in the pre-stapedotomy and post-stapedotomy FE models for 15 subsequent frequencies

Measurement frequency, $f$ (kHz)	A_in pre-stapedotomy (m)	A_in post-stapedotomy (m)	Measurement frequency, $f$ (kHz)	A_in pre-stapedotomy (m)	A_in post-stapedotomy (m)
0.40	1.64$\,\cdot \,$10$^{-8}$	2.93$\,\cdot \,$10$^{-8}$	2.50	2.84$\,\cdot \,$10$^{-9}$	3.48$\,\cdot \,$10$^{-9}$
0.50	1.53$\,\cdot \,$10$^{-8}$	2.62$\,\cdot \,$10$^{-8}$	3.15	6.40$\,\cdot \,$10$^{-10}$	9.10$\,\cdot \,$10$^{-10}$
0.63	1.90$\,\cdot \,$10$^{-8}$	1.62$\,\cdot \,$10$^{-8}$	4.00	7.73$\,\cdot \,$10$^{-10}$	3.35$\,\cdot \,$10$^{-10}$
0.80	1.97$\,\cdot \,$10$^{-8}$	1.89$\,\cdot \,$10$^{-8}$	5.00	8.36$\,\cdot \,$10$^{-10}$	2.62$\,\cdot \,$10$^{-10}$
1.00	1.02$\,\cdot \,$10$^{-8}$	1.05$\,\cdot \,$10$^{-8}$	6.30	6.14$\,\cdot \,$10$^{-10}$	1.59$\,\cdot \,$10$^{-10}$
1.25	8.92$\,\cdot \,$10$^{-9}$	7.31$\,\cdot \,$10$^{-9}$	8.00	1.69$\,\cdot \,$10$^{-10}$	1.43$\,\cdot \,$10$^{-10}$
1.60	7.12$\,\cdot \,$10$^{-9}$	8.58$\,\cdot \,$10$^{-9}$	10.00	7.57$\,\cdot \,$10$^{-11}$	1.85$\,\cdot \,$10$^{-10}$
2.00	4.93$\,\cdot \,$10$^{-9}$	6.25$\,\cdot \,$10$^{-9}$			

### FE simulation of RW membrane vibration following stapedotomy surgery

After validation, the FE model was used to investigate the effect of piston prosthesis implantation on the RW vibration.

**Fig. 7:**
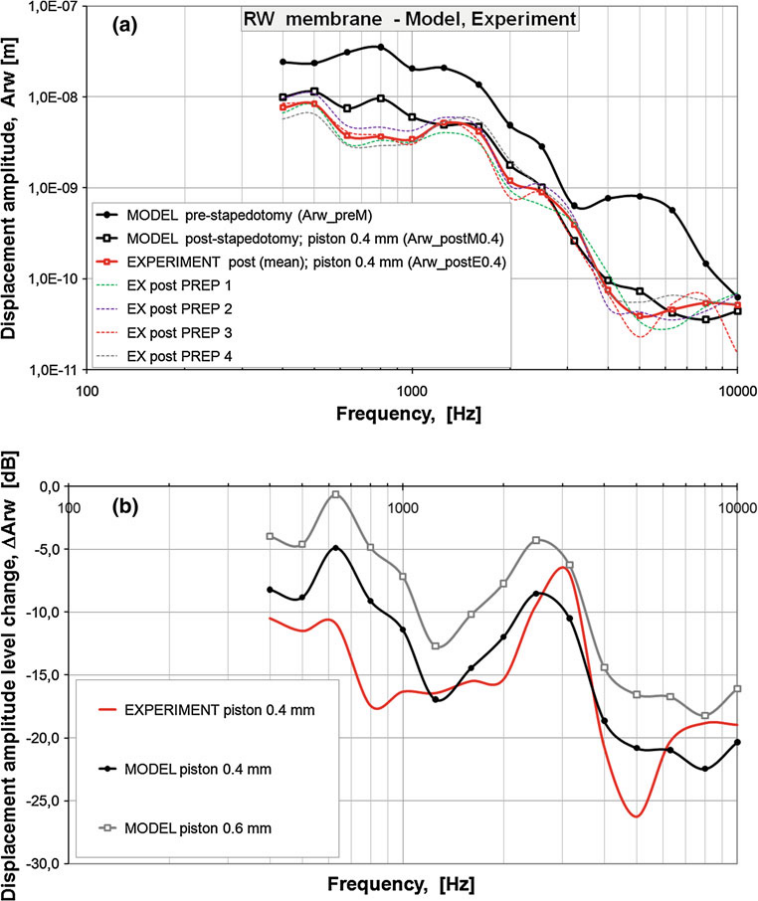
**a** Post-stapedotomy amplitude frequency profiles of the RW membrane displacement (Arw) while the 0.4-mm piston stapes prosthesis was inserted into the oval window. The figure presents the model-derived frequency response curve (MODEL post-stapedotomy, *thick, black, solid curve line, white squares*) while the amplitudes of the harmonic displacement excitations are given in Table 2 (A_in post-stapedotomy) in comparison with the corresponding curves (EX post SPECIMEN, *thin, colored, dashed curve lines*) obtained from four temporal bone specimens in the post-stapedotomy state when 90 dB SPL pure tones were applied in the external ear canal, and the values of average displacement amplitude of four points for all specimens (EXPERIMENT post(mean), *thick, red, solid curve line*). The model-derived pre-stapedotomy amplitude frequency profile of the RW membrane displacement (MODEL pre-stapedotomy, *thick, black, solid curve line, black circles*) was shown to compare with the post-stapedotomy profiles. **b** Changes in the model-derived RW membrane displacement amplitude ($\Delta$Arw, where the magnitude of $\Delta$Arw equals 20*log$_{10}$[Arw$_{\mathrm{post}}$/Arw$_{\mathrm{pre}}$]) induced by the 0.4-mm piston stapes prosthesis (MODEL piston 0.4 mm, *black, solid curve line*), the 0.6-mm piston stapes prosthesis (MODEL piston 0.6 mm, *gray, solid curve line*) and measured before and after experimental stapedotomy with the 0.4-mm piston prosthesis (EXPERIMENT piston 0.4 mm, *red, solid curve line*). For the model *curves*, the amplitudes of the harmonic displacement excitations are given in Table 2

Firstly, a piston prosthesis with a diameter of 0.4 mm was considered. Figure 7a shows the frequency response curves of the RW membrane displacement derived from the FE post-stapedotomy model (Arw_postM0.4) and control experimental curves measured from the human temporal bones following the stapedotomy surgery (Arw_postE0.4) compared to the curve for the FE pre-stapedotomy model. As can be seen, the FE model shows a maximum reduction in RW membrane displacement amplitude for the post-stapedotomy state within the 4–8 kHz frequency range. The post-stapedotomy FE model displacement amplitude curve (Arw_postM0.4) falls well within the range of the four temporal bone control experimental curves for frequencies above 1.25 kHz. Compared with the control experimental curves, the post-stapedotomy FE model showed higher values for the RW membrane displacement amplitudes for frequencies from 0.5 to 1.0 kHz. However, in general, the simulation from the post-stapedotomy FE model shows a pattern similar to the mean experimental curve.

Figure 7b shows the 0.4- and 0.6-mm piston-induced changes in the RW membrane displacement amplitude ($\Delta$Arw, where the magnitude of $\Delta$Arw equals 20*log$_{10}$[Arw$_{\mathrm{post}}$/Arw$_{\mathrm{pre}}$]). The model-derived magnitudes of $\Delta$Arw across the pre-stapedotomy and the 0.4-mm piston post-stapedotomy state were 8.8, 9.1, 14.4, 10.5, 18.6, 20.8, 21.0, and 22.5 dB at frequencies of 0.5, 0.8, 1.6, 3.15, 4.0, 5.0, 6.3, and 8 kHz, respectively. The mid- and high-frequency decrease in the magnitude of Arw has similar features to the mid- and high-frequency hearing loss observed in patients undergoing stapedotomy.

**Fig. 8:**
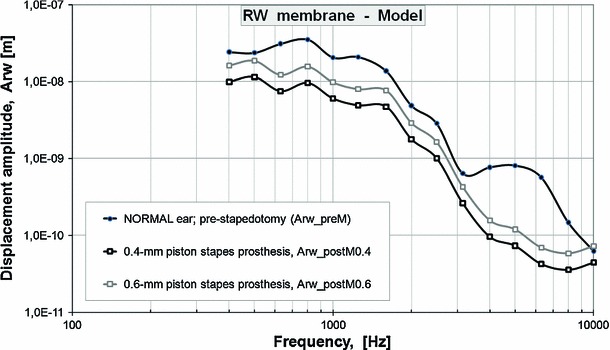
Pre-stapedotomy and post-stapedotomy model-derived amplitude frequency profiles of the RW membrane displacement (Arw). The figure presents the pre-stapedotomy frequency response curve (Arw_preM, NORMAL ear, *thick, black, solid curve line, black circles*) while the amplitudes of the harmonic displacement excitations are given in Table 2 (A_in pre-stapedotomy) in comparison with the post-stapedotomy curves while the 0.4-mm piston stapes prosthesis (Arw_postM0.4, 0.4-mm, *thick, black, solid curve line, white squares*) and 0.6-mm piston stapes prosthesis (Arw_postM0.6, 0.6-mm, *thick, gray, solid curve line*) were inserted into the oval window. The model-derived post-stapedotomy amplitude frequency profile of the RW membrane displacement was obtained for the amplitudes of the harmonic displacement excitations given in Table 2 (A_in post-stapedotomy)

After simulation for the 0.4-mm piston prosthesis, the frequency response curves of the RW membrane displacement were derived from the FE post-stapedotomy model for the 0.6-mm piston prosthesis and the new stapes prosthesis currently under investigation at Warsaw University of Technology. Figure 8 presents the model-derived frequency response curves for the post-stapedotomy state using the 0.4- and 0.6-mm piston stapes prosthesis compared to the curve for the FE pre-stapedotomy state (normal ear).The model-derived displacement amplitude difference across the curves for 0.4 and 0.6-mm piston prosthesis was approximately 4 dB at all frequencies. However, both post-stapedotomy model-derived frequency response curves were still below the curve for the normal ear.

## Discussion

At present, the piston stapes prosthesis is the most commonly used prosthesis replacing an immobilized stapes in cases of otosclerosis. However, the majority of patients report a lack of satisfactory hearing outcomes following the stapes surgery, especially for frequencies above 2 kHz. This leads to an in-depth analysis of the process of sound conduction through the inner ear structures and to an attempt to develop a new type of stapes prosthesis providing a sufficient level of excitation to the vibrations of perilymphatic fluid.

**Fig. 9:**
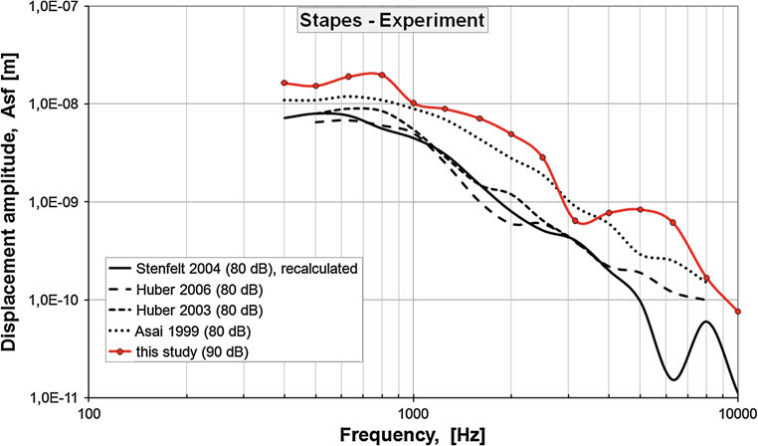
Comparison of stapes displacement amplitudes between Asai et al. (1999, 80 dB SPL at the TM), Huber et al. (2003 and 2006, 80 dB SPL at the TM), Stenfelt et al. (2004a, 80 dB SPL at the TM), and the present study (90 dB SPL at the TM). The velocity amplitudes reported by Stenfelt et al. (2004a) were recalculated to obtain the displacement amplitudes according to the formula A$$_{\mathrm{displacement}}$$ = A$$_{\mathrm{velocity}}$$/cos(2*3.14*$$f)$$ where A$$_{\mathrm{velocity}}$$—velocity amplitude, $$f$$—frequency

**Fig. 10:**
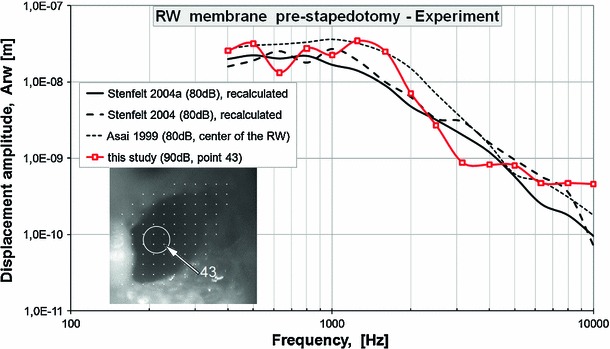
Comparison of round window displacement amplitudes for the pre-stapedotomy state between Asai et al. (1999, Fig. 5, center of the RW, 80 dB SPL at the TM), Stenfelt et al. (2004a, Fig. 4c, the *highest curve*, 80 dB SPL at the TM), Stenfelt et al. (2004b, Fig. 3a, the *highest curve*, 80 dB SPL at the TM), and the present study (scan point 43 on the RW membrane, 90 dB SPL at the TM). The velocity amplitudes reported by Stenfelt et al. (2004a, b) were recalculated to obtain the displacement amplitudes according to the formula A$_{\mathrm{displacement}}$ = A$_{\mathrm{velocity}}$/cos(2*3.14*$f$) where A$_{\mathrm{velocity}}$—velocity amplitude, $f$—frequency

The results of our modeling and experimental studies showed the differences in the RW vibrations between physiological conditions and following stapedotomy. The motion of the RW membrane has been used to determine the efficiency of the transfer of acoustic energy to the inner ear and to predict hearing results following some types of middle ear reconstructions (Asai et al. 1999; Mehta et al. 2003; Stenfelt et al. 2004b; Chien et al. 2007). This justifies the experimental and modeling investigations of the RW vibration in the case of stapedotomy surgery. To develop an appropriate simulation model of cochlear macromechanics, knowledge of the vibration parameters has been required, based on the experimental measurements. We used the LDV technique to find the displacement amplitude of the SF, the piston of the stapes prosthesis, and the RW membrane from fresh cadaver temporal bone specimens. We do not publish the phase data because our data differ substantially from the earlier studies (e.g., Stenfelt et al. 2004a). We suspect that the experimental phase data obtained by us are not correlated in time with the sound signal supplied to the external ear canal. Therefore, the analysis of the phase data without repeating the measurements is impossible. Nevertheless, our measurement results of the displacement amplitudes were comparable with other researchers’ studies (Asai et al. 1999; Huber et al. 2003, 2006; Stenfelt et al. 2004a, b). Figure 9 compares our measurement results of the stapes displacement amplitude to Fig. 5 of Asai et al. (1999), Fig. 6C of Huber et al. (2003), Fig. 2 of Huber et al. (2006), and Fig. 4a of Stenfelt et al. (2004a). Stenfelt et al. (2004a) reported the velocity amplitude of points on the stapes footplate. We recalculated these data to obtain the displacement amplitude (Asf) according to the formula Asf = Vsf/cos(2*3.14*$f$) where Vsf—velocity amplitude, $f$—frequency. Asai et al. (1999), Huber et al. (2003, 2006), and Stenfelt et al. (2004a) performed the experiments when a sound stimulation of 80 dB SPL was presented at the tympanic membrane. Because we measured the stapes displacement at 90 dB SPL, our measurement results are slightly higher compared to others. Figure 10 shows comparison of the RW displacement amplitude for the pre-stapedotomy state between (Asai et al. 1999, Fig. 5, center of the RW), Stenfelt et al. (2004a, Fig. 4c, the highest curve), Stenfelt et al. (2004b, Fig. 3a, the highest curve), and the present study. All curves are similar, but it should be noted that our measurement was made with 90 dB SPL. However, because complicated vibration patterns of the RW, comparing the RW membrane vibration at a single point, can give erroneous results. To our knowledge, only Stenfelt et al. (2004b) measured the RW vibration for the post-stapedotomy state. Stenfelt et al. made their measurement for a 0.6-mm piston stapes prosthesis with a sound pressure level at the tympanic membrane of 80 dB SPL. The results from nine target positions closed to the center of the RW membrane were presented as relative measures (Fig. 6, Stenfelt et al. 2004b). Therefore, it is difficult to compare our measurement results of the RW displacement amplitude (Arw) for a 0.4-mm piston stapes prosthesis and 90 dB SPL to the measurement results reported by Stenfelt et al. (2004b). In our measurement, the piston prosthesis substantially changed the vibration pattern of the RW membrane for the entire frequency range 0.5–10 kHz (Wysocki et al. 2011). Similar results were recorded by Stenfelt et al. (2004b). This suggests that the fluid volume displacement at the RW should be calculated to estimate the stimulation of the cochlea, especially in the case of stapedotomy surgery.

As has been demonstrated in the literature, the fresh temporal bone preparation is a good model for preclinical, in vitro studies, and laser Doppler vibrometry is a suitable tool for assessing middle ear prosthesis performance. Passive macromechanics of the hearing organ in fresh cadaver temporal bones functions similarly to physiological conditions provided that the temporal bone specimens are harvested from human cadavers within 48 h of death, protected from drying and stored without freezing until measurement, and the measurements are taken within 1–6 days of death (Aibara et al. 2001; Rosowski et al. 1990; Goode et al. 1993).

Our temporal bone preparation enabled us to make control measurements of the round window membrane vibration before and after experimental stapedotomy. We used the RW membrane displacement amplitudes (Arw) as an index of sound delivery to the cochlear partition. This assumption is similar to those made by Kringlebotn and Gundersen (1985), Kringlebotn (1995), Asai et al. (1999), Mehta et al. (2003), Stenfelt et al. (2004a, b), Chien et al. (2007). The differences between Arw for the pre-stapedotomy and post-stapedotomy states can be treated as an incomplete closure of the air–bone gap resulting in conductive hearing loss in patients undergoing stapedotomy.

The stapedotomies that we performed in our temporal bones were all relatively similar in piston diameters (0.4 and 0.6 mm) and were at similar locations (piston in the center of the SF, ribbon around the long process of the incus) to the clinical procedures. It would be possible in future studies to use the temporal bone preparations to systematically examine varying dimensions of the new stapes prosthesis in an attempt to determine whether these dimensions affect the resulting hearing outcomes.

In all four bones, the Arw magnitude was decreased in response to piston insertion in the SF, in particular at frequencies exceeding 2 kHz (Fig. 7b). This is consistent with the mid- and high-frequency air–bone gap observed in patients undergoing stapedotomy. The reduction in Arw magnitude by a piston prosthesis is also consistent with the approximately fivefold lower stimulation of perilymph vibration in response to air-conducted stimulus compared to physiological situation (Stenfelt et al. 2004b; Wysocki et al. 2011).

The objective of this study was to assess the influence of stapedotomy surgery on the round window membrane vibration and to estimate the postoperative outcomes using the finite element (FE) method. We have presented a 3D FE cochlear model to predict the RW membrane vibrations induced by the normal SF, the 0.4-mm piston prosthesis, and the 0.6-mm piston prosthesis. Due to the highly complex geometry of the cochlea in the FE model presented, the cochlear curvature, the Reissner’s membrane and the micromechanical structure of the organ of Corti are omitted, and the scala media is included in the SV. We also assumed that the cochlea is detached from the middle ear structures and that stimulation of the perilymph occurs by applying the vibrations to the oval window. These simplifying assumptions have been accepted by many authors for investigating the cochlear macromechanics using mathematical, FE, and physical models (Andoh and Wada 2004; Kolston and Ashmore 1996; Lechner 1993; Neely 1981; Steele and Lim 1999; Watts 2000; Wittbrodt et al. 2006; Gan et al. 2007). All of these models assumed that stimulation of the cochlea occurs by applying the vibrations to the oval window or to the one side of the scala vestibule through the SF.

Most of the experiments were performed to investigate the physiological motion of the SF. It has been reported that the physiological motion of the SF in response to acoustic stimulation is predominantly piston-like at low frequencies and includes rocking around the short and long axes of the SF at high frequencies (Heiland et al. 1999; Hato et al. 2003; Sim et al. 2010). Nevertheless, most current human cochlear models do not consider the effects of this rocking motion because it is believed not to contribute to fluid volume displacement at the oval window and to have negligible effects on cochlear macromechanics (Kolston and Ashmore 1996; Lim and Steele 2002; Shera et al. 2005; Pozrikidis 2007, 2008). The same assumption has been made for this study and both the SF and the piston prosthesis motion were assumed as piston-like. In our FE cochlear model, the stimulation of the perilymph was assumed as real measured amplitude vibrations of both the stapes in the physiological state and the stapes piston prosthesis in the post-implantation state. This assumption takes into account the change in the cochlear input impedance resulting from interactions between the cochlea and the normal or implanted middle ear. It was shown experimentally that a small-fenestra stapedotomy acts to decrease the impedance that loads the ossicular chain (Rosowski et al. 2003), because of difference in area between the normal SF and the piston of the stapes prosthesis. Moreover, stapedotomy bypasses the impedance of the AL which is replaced by an unknown impedance of the seal around the piston (we used a blood clot as in clinical situations). These two factors (the area ratio and bypassing of the AL impedance) together change the impedance imposed by the inner ear on the middle ear such that equal sound pressures levels in the external ear canal produce different vibrations of the prosthesis compared with normal stapes motions. To predict hearing results after stapedotomy surgery using different stapes prostheses, the FE model requires an adjustment of the input displacement amplitudes (A_in post-stapedotomy). This is the main difficulty in this approach.

Another issue is the lack of mechanical properties of the cochlear soft tissues, such as the BM, the RW membrane, and the AL, obtained from experimental measurements. In view of the above, we have initially assumed the values for mechanical parameters given by Gan et al. (2007). These parameters were then checked in the validation process by comparing the RW membrane displacement amplitudes (Arw) derived from the FE model with the experimental data obtained from temporal bone experiments for the pre-stapedotomy state. A similar approach has been used by many authors (Ferris and Prendergast 2000; Sun et al. 2002; Koike et al. 2002; Gan et al. 2004, 2007). After the validation process, the density of the AL and all mechanical properties of the perilymph fluid, the BM, and the RW membrane were left unchanged (Table 1). For the AL, the Young’s modulus of 5.5 MPa proved to be too high, resulting in a large reduction in the RW displacement amplitude, especially for frequencies below 3 kHz. The stiffness of the AL was predicted to reduce the vibration amplitude of the stapes (Kringlebotn 1988; Ferris and Prendergast 2000), thereby reducing the RW vibration amplitude. To minimize the difference between the FE model and experimental data, the Young’s modulus for the AL in our FE model was decreased to 0.2 MPa. This value is the same as assumed by Gan et al. (2006). Also, the Raleigh damping parameters $\alpha =0\,\text{ s}^{-1}$ and $\beta =0.00005$ s for both the AL and the SF were then determined by the validation process. The final values of the mechanical properties used in our FE model are listed in Table 1. The FE model-predicted displacement amplitude of the RW (Arw_preM) for the pre-stapedotomy state is plotted with black line in Fig. 5, and it can be seen that the results match the experimental data in the frequency range of 0.5–7 kHz. At higher frequencies, Arw_preM lies below the experimental curves, likely due to the complex high-frequency three-dimensional motion of the stapes (Heiland et al. 1999; Hato et al. 2003). Our FE model assumes the values of input displacement amplitudes (A_in) in the pre-stapedotomy state (Fig. 4, red line), which may be different from the normal physiological state. The A_in pre-stapedotomy amplitudes were applied to all stapes footplate nodes in the direction perpendicular to the footplate. The rocking motion of the stapes was neglected. The values of A_in amplitudes were taken as equal to the displacement amplitudes measured on the stapes head. This assumption is completely correct only below 2 kHz. Above 2 kHz, a rocking motion increases logarithmically with frequency (Heiland et al. 1999). In this frequency range, measurement of stapes head may represent vibration only at the center of the footplate. For frequencies above 7 kHz, the fluctuations of the rocking ratio (Heiland et al. 1999), defined as the maximum difference in displacement between the anterior and posterior footplate to displacement at the center of the stapes footplate, correspond to the fluctuations of the pressure in both the scala vestibuli and scala tympani reported by Nakajima et al. (2008; Fig. 3). This suggest that in this frequency range, complex footplate motion may induce higher volume velocity compared to piston footplate motion with velocity measured at the center of the footplate. The scala tympani pressure and volume velocity at the RW have a significant effect on the vibration amplitudes of the RW membrane. This may explain the discrepancy of the FE model results and experiments in our Fig. 5 for the pre-stapedotomy state.

In the post-stapedotomy state with the 0.4-mm piston prosthesis, the RW membrane displacement amplitudes derived from our FE cochlear model (Arw_postM0.4) were compared with the data measured from the cadaver temporal bones (Arw_postE0.4) (Fig. 7a). The FE model predicted that the piston stapes prosthesis implantation would affect the RW membrane displacements, which is consistent with the experimental data reported by Wysocki et al. (2011) and Stenfelt et al. (2004b). This leads to the conclusion that the simulation results derived from our 3D FE model are reliable. The value of displacement reduction was related to the piston diameter, and the maximum reduction was achieved at the highest frequencies, such as those exceeding 3 kHz in this study. The reduction in displacement amplitude increases with the increase in frequency and can be treated as an incomplete closure of the preoperative air–bone gap resulting in conductive hearing loss following stapedotomy.

To further investigate the effects of our new stapes prosthesis on sound transmission through the inner ear, we conducted a preliminary simulation of the RW frequency–displacement curve. The encouraging simulation results showed that the new stapes prosthesis should be subjected to further detailed investigations, both theoretical and experimental. The study reported here is considered as a step toward the potential engineering applications of the FE model in optimizing new stapes prosthesis.

## Conclusion

The vibration amplitudes of the RW membrane before and after stapes prosthesis implantation can be used to predict the effect of stapedotomy on sound transmission through the inner ear. This study indicates that stapedotomy surgery can be well simulated using the FE cochlear model to predict the hearing results obtained by various types of stapes prostheses. The FE model is suitable for studying the dynamic behaviors of the normal and post-stapedotomy inner ear and potentially useful for designing and testing new stapes prosthesis. Some of our future investigations will focus on identifying such geometric parameters of the new stapes prosthesis which will ensure a near-to-normal hearing restoration, especially after stapedotomy surgery.

## Abbreviations

AL: Annular ligament
A_in: Input displacement amplitude
A_rw: Displacement amplitude of the round window membrane
A_rw_postE0.4: Displacement amplitude of the round window membrane measured from the temporal bone following the stapedotomy surgery with the 0.4-mm piston prostesis
A_rw_postM0.4: Displacement amplitude of the round window membrane derived from the FE post-stapedotomy model with the 0.4-mm piston prosthesis
A_rw_postM0.6: Displacement amplitude of the round window membrane derived from the FE post-stapedotomy model with the 0.6-mm piston prosthesis
A_rw_preE: Displacement amplitude of the round window membrane measured from the temporal bone in the normal physiological state (pre-stapedotomy)
A_rw_preM: Displacement amplitude of the round window membrane derived from the FE pre-stapedotomy model
$\Delta$Arw: Change in the round window membrane displacement amplitude between the pre-stapedotomy and the post-stapedotomy states
BM: Basilar membrane
f: Frequency
RW: Round window
SF: Stapes footplate
SPL: Sound pressure level
ST: Scala tympani
SV: Scala vestibuli
